# Supplementary material for: Deletion of wfs1 Impairs Oligodendrocyte Precursor Cells Dorsal Distribution and Myelination Through the wfs1-hmgcs1 Axis in Zebrafish
Source: Biology (Basel). 2026 Aug 16;15(16):1402. doi: 10.3390/biology15161402 (PMC13510151; doi:10.3390/biology15161402)
Supplement: Supplementary file 1 [file biology-15-01402-s001.zip › File S1-Transcriptome sequencing data and differentially expressed genes (DEGs) in wfs1-- zebrafish at 5 dpf/2.KEGG/wfs1KOvsWT/DOWN/src/dre00983.html]

dre00983


- dre:794381

- Down regulated genes

794381(-1.83200406616465)

- dre:794381

- Down regulated genes

794381(-1.83200406616465)

- dre:103910413

- Down regulated genes

103910413(-1.41996191255597)

- dre:100005763

- Down regulated genes

100005763\_1(-1.3448133369938)

- dre:437016

- Down regulated genes

437016\_1(-4.13489163251683)

- dre:103910413

- Down regulated genes

103910413(-1.41996191255597)

- dre:492517

- Down regulated genes

492517\_1(-4.29681008078085)

- dre:406731

- Down regulated genes

406731(-3.65881284610119)
- dre:678597

- Down regulated genes

100415796(-3.10645904576552)
- dre:570913

- Down regulated genes

570913\_1(-2.18577817117659)
- dre:767756

- Down regulated genes

767756(-1.81780175682628)

- dre:794381

- Down regulated genes

794381(-1.83200406616465)

- dre:406703

- Down regulated genes

406703(-2.80714548639507)

Close
